# Abundant Cyanobacteria in Autumn Adhering to the Heating, Ventilation, and Air-Conditioning (HVAC) in Shanghai

**DOI:** 10.3390/microorganisms11071835

**Published:** 2023-07-19

**Authors:** Changliang Nie, Xueyun Geng, Runqi Zhang, Lina Wang, Ling Li, Jianmin Chen

**Affiliations:** 1 Shanghai Key Laboratory of Atmospheric Particle Pollution and Prevention (LAP3), Department of Environmental Science & Engineering, National Observations and Research Station for Wetland Ecosystems of the Yangtze Estuary, Fudan University, Shanghai 200438, China; niechangliang@fudan.edu.cn (C.N.);; 2 IRDR International Center of Excellence on Risk Interconnectivity and Governance on Weather/Climate Extremes Impact and Public Health, Institute of Atmospheric Sciences, Fudan University, Shanghai 200438, China; 3 Institute of Eco-Chongming (IEC), Shanghai 200062, China

**Keywords:** season, cyanobacteria, cyanosphere, indoor environment, heating, ventilation, air-conditioning (HVAC)

## Abstract

Cyanobacteria are ever-present, mainly flourishing in aquatic environments and surviving virtually in other habitats. The microbiota of indoor dust on the pre-filter of heating, ventilation, and air-conditioning (HVAC) systems, which reflect indoor microbial contamination and affect human health, has attracted attention. Contemporary studies on cyanobacteria deposited on the pre-filter of HVAC remain scant. By the culture-independent approach of qPCR and high throughput sequencing technologies, our results documented that the cyanobacterial concentrations were highest in autumn, occurred recurrently, and were about 2.60 and 10.57-fold higher than those in winter and summer. We proposed that aquatic and terrestrial cyanobacteria contributed to the pre-filter of HVAC by airborne transportation produced by wave breaks, bubble bursts, and soil surface by wind force, owing to the evidence that cyanobacteria were commonly detected in airborne particulate matters. The cyanobacteria community structure was characterized in Shanghai, where Chroococcidiopsaceae, norank_cyanobacteriales, Nostocaceae, Paraspirulinaceae, and others dominated the dust on the pre-filter of HVAC. Some detected genera, including *Nodularia* sp., *Pseudanabaena* sp., and *Leptolyngbya* sp., potentially produced cyanobacterial toxins, which need further studying to determine their potential threat to human health. The present work shed new insight into cyanobacteria distribution in the specific environment besides aquatic habitats.

## 1. Introduction

Algae are a class category mainly grown in water bodies such as ponds, lakes, rivers, and oceans, and most studies are aimed at harmful algae control [1] and utilization [2,3,4]. Cyanobacteria are one of the important classes, as they are ubiquitous, and parts contain toxic compounds such as microcystins, which aroused much attention [5]. However, besides the aquatic environment, cyanobacteria exist in other habitats, e.g., terrestrial, clouds, rain, and airborne environments [6,7,8]. Recently, bioaerosol attracted considerable attention owing to the COVID-19 epidemic [9]. Likewise, cyanobacteria aerosolization received considerable attention due to making widespread exposure possible, concerning, and threatening to human health [10,11,12,13,14,15,16]. One study indicated that exposure to aerosolized harmful algal bloom (HAB) particles resulted in health risks, both in the long and immediate term [17]. The fate of bioaerosol, including airborne microbiota concentration and community structure, is commonly influenced by specific land use (e.g., wastewater treatment [18] and vegetable plastic greenhouses [19]) and ambient environmental factors (e.g., metrological parameters [20], air pollution [21], and season change cycle [22]). The outdoor bioaerosols mainly contributed to airborne microbiota in the household [23]. The heating, ventilation, and air-conditioning (HVAC) filters could be a viable option for estimating indoor biological contaminants [24]. The HVAC systems significantly impact the indoor environment, and HVAC systems contaminated by microorganisms can cause severe indoor air quality problems [25]. Low indoor air quality can harm human health because people tend to spend more than 80% of their time in the indoor environment, suggesting that it is urgent to understand the microbiota in case of unexpected health risks caused by microbial contamination [26,27]. Contemporary research mainly focuses on bacteria and fungi [28]. Most research revealed that the microbial community structure in HVAC systems was dominated by Proteobacteria, Actinobacteria, Firmicutes, Cyanobacteria, and others [25]. Cyanobacteria were usually detected, and occupied a proportion, but lacked further systematic analysis.

The algae that, by airborne transportation, attached to buildings or even indoor furniture was ubiquitous, and the increasing studies realized that their links potentially accelerated the degradation of building materials [29,30]. The airborne cyanobacteria can enter the household. The indoor HVAC was a spot for cyanobacteria deposition by airborne transportation, which also could be the source of cyanobacteria emission when air conditioners were operating and might cause cyanobacteria-associated risks to human health [27]. Similar to the bioaerosol, the characteristics of the airborne cyanobacteria are also affected by geographic factors, which means specific cities will shape the different results. Shanghai is an international megacity with a large population of 26 million which determines the wide usage of HVAC systems. Additionally, Shanghai is located east of China, which is adjacent to the East China Sea and has many inland ponds, lakes, and rivers. What those geographic features will affect the cyanobacteria on the pre-filter of HVAC is interesting. Thus, estimating cyanobacteria in dust from the HVAC is significant. Another point worth considering is that our work will bridge the gap in understanding the cyanobacteria existence in specific habitats, as the studies on cyanobacteria associated with HVAC remain understudied. Furthermore, the cyanobacterial variation follows a seasonal time cycle variation. Climate changes, including increasing temperature [28], humidity, and wind speed or direction, can determine the cyanobacterial community structure, further affecting the aquatic or terrestrial algal distribution [31]. To a large extent, seasonal changes play the same role in climate change, determining the ambient environmental factors, such as temperature, humidity, wind speed, wind direction, and others, directly shaping the bioaerosol distribution [22]. To our knowledge, research on the characteristics of the cyanobacteria community on the pre-filter of HVAC under the season cycle is still sparse, and the estimation of cyanobacteria response to the season variation in the dust of HVAC is meaningful.

The previous study mostly used the culture-dependent approach and observation by microscopy to study the airborne cyanobacteria distribution and their relationship between the building facades [32]. The mentioned approach could provide limited information on the cyanobacteria community and concentration on HVAC. High-throughput technologies revealed more information on cyanobacteria distribution in different environments [33] and were employed to unveil the microbial community structure in cyanobacteria-laden drinking water sludge storage [34]. Therefore, the present work was conducted to analyse the cyanobacteria distribution in the dust on the pre-filter from HVAC in Shanghai across the seasons (i.e., autumn in 2021, winter in 2022, summer in 2022, and autumn in 2022). The present work aimed to answer the cyanobacterial concentration and community structure in dust from the HVAC system and estimated the diversity and difference of cyanobacteria under different seasons, which can shed new understanding on the cyanobacteria distribution in the indoor dust of HVAC.

## 2. Materials and Methods

### 2.1. Sample Collection and Pretreatment

The HVAC filters were collected in different households in Shanghai, as follows: six samples in October 2021 as autumn_2021, five samples in December 2021 as winter_2021, nine samples in August 2022 as summer_2022, and seven samples in October 2022 as autumn_2022. The filters were directly removed from domestic air-conditioning systems. Sterilized polythene bags were employed to hold filters immediately, transported back to the lab, and stored at −80 °C before further pretreatment. Regarding the pretreatment of samples, filters were cut into pieces and stored using 50 mL sterilized tubes. The pretreated sample was extracted by 40 mL autoclaved water and vigorous vortexing for 20 min (Vortex genie-2, Scientific Industries Co., Ltd., Bohemia, NY, USA). Next, the filters in the washed suspension were discarded, followed by centrifugation at 4500 rpm for 20 min, and the upper supernatant was discarded except for 1.5 mL. The lower layer was re-suspended and immediately transferred into a 2 mL autoclaved tube, then the tube was centrifuged at 12,000 rpm, and the supernatant was discarded. All dust samples were stored at −80 °C until further processes.

### 2.2. DNA Extraction and Miseq Sequencing

DNA extraction was performed based on the manufacturer’s guidelines, as recommended by the E.Z.N.A.^®^ soil DNA Kit (Omega Bio-tek, Norcross, GA, USA). The concentration and quality of DNA were determined by 1.0% agarose gel electrophoresis and a NanoDrop^®^ ND-2000 spectrophotometer (Thermo Scientific Inc., Waltham, MA, USA) and kept at −80 °C. Regarding the high throughput sequencing process, the hypervariable V3–V4 region of the bacteria 16S ribosomal DNA genes was amplified by polymer chain reactions (PCR) using primers 338F and 806R. The bacterial 16S rRNA gene was amplified in triplicate. The 20 μL mixture for each amplification included 4 μL of 5× FastPfu Buffer, 0.4 μL of FastPfu Polymerase, 0.8 μL of each primer (5 μM), 2 μL of 2.5 mM dNTPs, 10 ng of template DNA, and ddH2O on an ABI GeneAmp^®^ 9700 PCR thermocycler (ABI, Los Angeles, CA, USA). The PCR process was initiated at 95 °C for 3 min, followed by 30 cycles of 95 °C for 30 s, 52 °C for 15 s, 72 °C for the 30 s, and a final extension at 72 °C for 5 min. Autoclaved water was carried out as a negative control for all PCR tests. Three replicates of PCR for each sample were combined. The final PCR products were separated by 2% agarose gel electrophoresis and purified using the AxyPrep DNA Gel Extraction Kit (Axygen Biosciences, Union City, CA, USA) according to the manufacturer’s instructions and quantified using Quantus™ Fluorometer (Promega, Madison, WI, USA). The purified products were stored at −80 °C until Illumina library construction. Sequencing libraries of the purified PCR products were prepared using the TruseqTM DNA Sample Prep Kit following the manufacturer’s instructions. Sequencing was performed on an Illumina MiSeq PE300 instrument (Illumina, San Diego, CA, USA) with the MiSeq reagent kit V3 (Illumina, San Diego, CA, USA) according to the standard protocols. 

### 2.3. Quantitative PCR Tests

Quantitative PCR (qPCR) was performed to quantify the bacterial gene copies per square centimetre of HVAC filters. The process was performed with the StepOnePlus™ real-time PCR detection system (Thermo). Each of the 20 μL volumes of the system contained 10 μL 2× TB Green Premix Ex Taq II (Takara), 0.8 μL of each primer (10 μM), 0.4 μL 50× ROX Reference Dye, 2 μL template DNA. Real-time PCR was initiated at 95 °C for 10 min, followed by 40 cycles of denaturation for 20 s at 95 °C, annealing for 30 s at 55 °C, and elongation for 30 s at 72 °C. Fluorescence signals were collected at 72 °C during PCR elongation. The 10-fold serial dilutions of plasmids (102 to 107 copies of the template) containing the 16S DNA obtained the standard curve to calculate the bacterial concentration. For each sample, three replicates for the qPCR test were used to quantify its cell level. All tests and experiments used autoclaved water without HVAC dust samples as a negative control. The cyanobacteria concentration was calculated by 16S rDNA copy number times their relative abundance [35].

### 2.4. Data Analysis

Operational taxonomic units (OTUs) were clustered with a 97% similarity cutoff [36,37] using UPARSE (version 7.1 http://drive5.com/uparse/, accessed on 1 January 2024.), and chimeric sequences were identified and removed. Subsequently, the OTUs were assigned to different taxonomic categories using the SILVA database. Alpha diversity, including observed species (sobs), Chao, and Shannon, were calculated to investigate the differences and variations in bacterial diversity and richness in HVAC filters by QIIME (V1.9.1).27.

## 3. Results

### 3.1. The Cyanobacteria Concentration and Abundance

**Figure 1:**
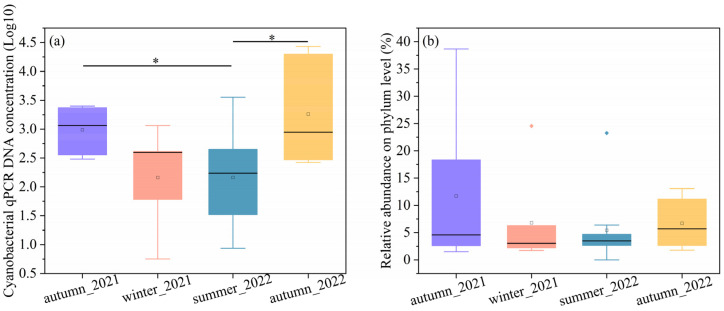
Represents the (**a**) cyanobacterial concentrations and (**b**) the relative abundance on the phylum level. * *p* < 0.05.

The concentration of DNA copy number and relative abundance can be used to profile the bacterial concentration [35]. The cyanobacteria concentrations were quantitatively shown by 16S rDNA copies multiplied by their relative abundance. To make a clear comparison, we took the logarithm of DNA copies, as shown in Figure 1a. The cyanobacterial DNA copies in both autumns were the highest and displayed similarly. The cyanobacterial concentrations in summer significantly differed from those in the two autumn seasons (*p* < 0.05). The cyanobacterial 16S rRNA gene concentrations ranged from 302.01 to 2506.01 copies/cm^2^ in autumn 2021, 5.66 to 1,148.75 copies/cm^2^ in winter 2021, 0 to 3556.05 copies/cm^2^ in summer 2022, and 266.03 to 27,065.00 copies/cm^2^ in autumn 2022. The median values of autumn were 1164.17 copies/cm^2^ in 2021 and 884.62 copies/cm^2^ in 2022, followed by winter (394.21 copies/cm^2^) and summer (96.93 copies/cm^2^). The median cyanobacteria DNA copies of atumun_2021 and autumn_2022 were about 12 and 9 times higher than summer values. 

The relative abundances of cyanobacteria compared to the total numbers of 16S rDNA sequences at the phylum level were shown in Figure 1b. The relative abundance of cyanobacteria at phylum varied significantly across the autumn samples. For example, the relative abundance of cyanobacteria ranged from 1.51% to 38.66% in autumn 2021. In comparison to the autumun_2021, fewer variations were found in the winter_2022, summer_2022, and autumn_2022 samples, with a range from 1.74% to 24.55%, 0 to 23.24%, and 1.77% to 13.07%, respectively. The results indicated that cyanobacteria are more abundant in the autumn than in summer and winter. The average relative abundances of cyanobacteria were as follows, autumn (11.71%) in 2021, winter (7.41%) in 2021, summer (5.40%) in 2022, and autumn (6.69%) in 2022. The median values were 4.57%, 2.26%, 4.41%, and 6.28%, corresponding to atumun_2021, winter_2021, summer_2022, and atumun_2022, respectively. 

### 3.2. The Cyanobacteria Community Diversity

**Figure 2:**
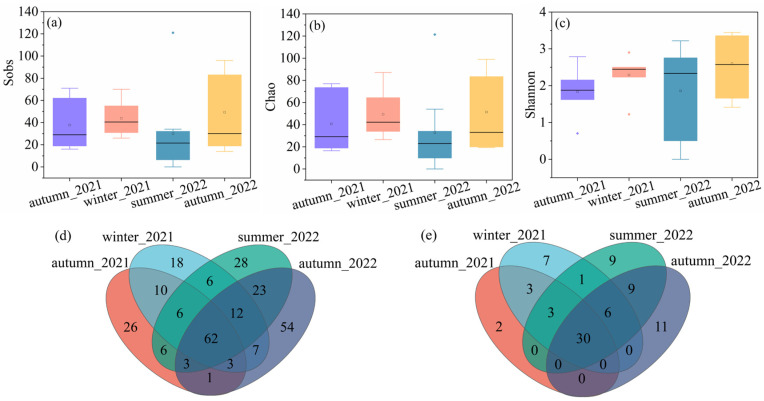
The alpha diversity of cyanobacteria community, (**a**) sobs, (**b**) chao, (**c**) Shannon, (**d**) Venn diagram on OTU level, and (**e**) Venn diagram on genus level.

Regarding the cyanobacterial diversity, the highest average values of sobs (43.83) and chao (49.41) were observed in winter_2021, followed by autumn_2022 (49.29 for sobs and 51.47 for chao), autumn_2021 (37.67 for sobs and 40.72 for chao), and summer_2022 (32.67 for sobs and 32.88 for chao). The average Shannon value was the highest in autumn_2022, with a value of 2.60, followed by winter_2021 (2.29), summer_2022 (1.86), and autumn_2021 (1.84). Figure 2d,e showed the Venn diagram characterizing the cyanobacterial genus distribution at OTU and genus levels. It indicated that 62 OTU and 30 genera co-existed in all the groups. The exclusive numbers of OTU were 26, 18, 28, and 54, corresponding to autumn in 2021, winter in 2021, summer in 2022, and autumn in 2022. The number of genus levels decreased compared to the OTU levels. The exclusive numbers of the genus were 2, 7, 9, and 11, corresponding to autumn in 2021, winter in 2021, summer in 2022, and autumn in 2022. Despite the varied values, no significant differences were observed in any of the samples.

### 3.3. The Difference in the Cyanobacteria Community

**Figure 3:**
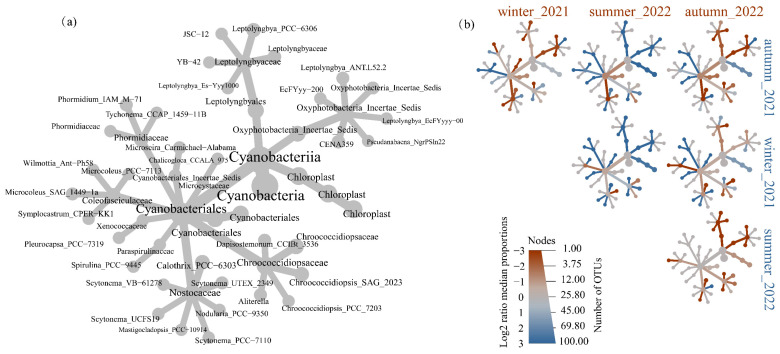
Tree-based visualizations for cyanobacteria taxa identified in samples. (**a**) The metacoder tree revealed the cladogram among the cyanobacteria across samples. (**b**) The heat tree revealed the pairwise comparisons of communities across different season samples. Node colour, node size, and edge colour represent the mean relative abundance, number of associated OTUs, and prevalence for each taxon across each sample.

The cyanobacterial community structures were depicted by a metacoder tree according to the top one hundred OTU in the collected samples, as shown in Figure 3. Figure 3a illustrated the phylogenetic relationships among the cyanobacteria. Figure 3b indicated the difference of taxa in specific sample groups. A phylogenetic signal was discernible within the specific seasonal samples amongst the enriched taxa. For example, the greater abundance of Leptolyngbyales was found in autumn_2021 and winter_2021, while it significantly lowered in summer_2022 and autumn_2022. Members of chloroplast were more abundant in autumn_2021 and winter_2021 than other samples. Nostocaceae were not significantly enriched in winter_2021, summer_2022, and autumn_2022. Xenococcaceae were not significantly enriched in autumn_2021 and winter_2021.

### 3.4. The Difference in the Cyanobacteria Community

**Figure 4:**
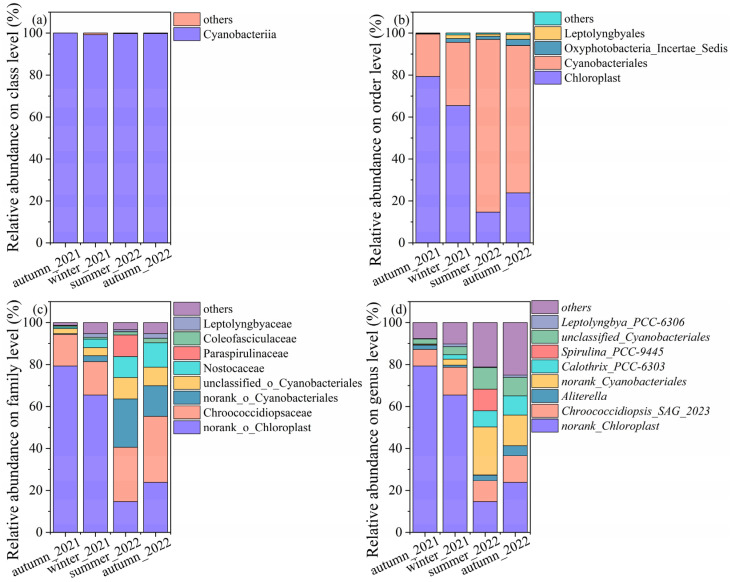
The relative abundance of cyanobacteria on the (**a**) class level, (**b**) order level, (**c**) family, and (**d**) genus level.

The relative abundances of cyanobacterial community structures at other levels were shown in Figure 4. Overall, cyanobacteria were predominant at the class level, which exceeded 99% across all the samples. At the order level, a proportion of genera in autumn_2021 and winter_2021 annotated to the chloroplast, with values of 79.29% and 65.46%, respectively, which induced the proportion of chloroplast-associated genus at the family and genus level. It accounted for 14.63% and 23.80% in summer_2022 and autumn_2022, respectively. The samples in the summer and autumn of 2022 were dominated by Cyanobacterales, with values of 82.31% and 70.23%, respectively. The lower relative abundances of Cyanobacterales were observed in autumn_2021 and winter_2021, with average values of 20.22% and 30.14%. Fractions were denoted to Oxyphotobacteria, Leptolyngbyales, and others. 

The results of the cyanobacteria community structure started to be versatile at the family level. The samples of autumn_2021 were dominated by norank_chloroplast (79.29%), Chroococcidiopsaceae (15.06%), unclassified_o_cyanobacteriales (2.42%), and others. Samples of winter_2021 were dominated by norank_chloroplast (65.47%), Chroococcidiopsaceae (15.92%), Nostocaceae (4.05%), unclassified_o_cyanobacteriales (2.42%), and others. The dominant genus, such as Chroococcidiopsaceae (31.51%), norank_chloroplast (23.80%), norank_Cyanobacteriales (14.48%), Nostocaceae (11.62%), and others were found in autumn_2022. The Chroococcidiopsaceae (25.93%), norank_Cyanobacteriales (23.01%), norank_chloroplast (14.63%), Paraspirulinaceae (10.32%), unclassified_Cyanobacteriales (10.23%), and others were predominant in summer_2022. 

Similarly, more taxa were annotated at the genus level. The relative abundances of cyanobacteria varied, even though one genus displayed different abundances in the same season in different years. The relative abundances of norank_Chloroplast were higher than other genera in autumn_2021 and winter_2022. The *Chroococcidiopsis* spp. accounted for a high proportion and were mainly categorized by three species. They were *Chroococcidiopsis*_SAG_2023, norank_*Chroococcidiopsaceae*, unclassified_*Chroococcidiopsaceae*, and *Chroococcidiopsis*_PCC_7203. *Chroococcidiopsis*_SAG_2023 were detected across all samples, as follows: winter_2022 (13.23%), autumn_2022 (12.63%), summer_2022 (9.90%), and autumn_2021 (7.91%). The second abundant taxon was norank_*Chroococcidiopsaceae*, with values of 2.15%, 9.85%, 9.31%, and 0.89%, corresponding to autumn_2021, autumn_2022, summer_2022, and winter_2022. *Chroococcidiopsis*_PCC_7203 and unclassified_Chroococcidiopsaceae were not rich and ranged from 0.09% to 1.53% and 0.42% to 2.86% across all the samples. The norank and unclassified cyanobacteriales ranged from 0.37% to 23.01% and 2.42% to 10.23%, indicating further identification in the following work. *Calothrix*_PCC-6303 dominated in summer_2022 and autumn_2022, with values of 7.72% and 9.16%, while they decreased to 0.14% and 2.14% in autumn_2021 and winter_2022. *Aliterella* accounted for 2.03%, 1.01%, 2.69%, and 4.88%, corresponding to autumn_2021, winter_2022, summer_2022, and autumn_2022, respectively. *Spirulina*_PCC-9445 was only detected in summer_2022, with a value as high as 10.32%. 

## 4. Discussion

Owing to limited research conducted on revealing the characteristics of cyanobacteria on the pre-filter of heating, ventilation, and air-conditioning (HVAC) systems, our work provided systemic results of cyanobacteria distribution and contributed to understanding the fate of the cyanobacteria community following the seasonal change cycle. 

**Figure 5:**
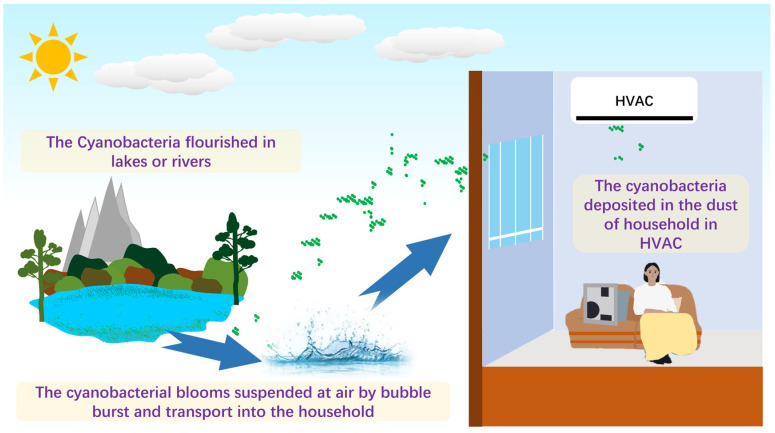
A scheme of potential source and transportation of cyanobacteria in HVAC.

As shown in Figure 5, an underlying mechanism was proposed. The water bodies, such as lakes, rivers, and oceans, can produce jet and film drops by wind or wave breaking [10], leading to two types of aerosol production. Sea spray aerosol (SSA) and lake spray aerosol (LSA) can occur, carrying the cyanobacteria from the aquatic environment to the atmosphere and enabling it to enter the household. That mechanism has been proved. May et al. (2018) found that the LSA was mainly composed of biological origin when the algal bloom happened [38]. The SSA and the LSA can produce many airborne particles composed of cyanobacteria, and that was the reason much of the research detected that airborne cyanobacteria adhere to airborne particle matters. For instance, Liu et al. (2018) indicated that Cyanobacteria were the second most abundant phylum in Hangzhou, with a value of 18% in all airborne particle matters [39]. Wei et al. (2020) found that Cyanobacteria accounted for the second abundant phylum of fine particle matter (PM_2.5_) in Jinan [21]. Hu et al. (2020) reported that the samples were characterized by abundant Cyanobacteria, which accounted for 30% (from 36% in the daytime to 25% in nighttime) [40]. Notably, the relative abundance of airborne cyanobacteria fluctuated significantly, and one can reach approximately 100% [40]. The apparent evidence indicated that airborne cyanobacteria accounted for different relative abundances, and airborne transportation was the vehicle for aquatic cyanobacteria entering the household.

The mentioned discussion confirmed that the aerosolized nature of the cyanobacteria contributed to their distribution in indoor dust on the pre-filter of heating, ventilation, and air-conditioning (HVAC) systems, which will be affected by various factors. The algal community structure generally follows a seasonal variation due to their growth subjected to ambient environmental factors [41]. Likewise, the bioaerosol-associated research also showed a seasonal mechanism [42]. Romano et al. (2020) found a higher relative abundance of cyanobacteria in winter than in spring [43]. Regarding the cyanobacteria concentration, we found that the cyanobacterial DNA copies in autumn exceeded other seasons. A similar trend was found in some local lakes. For instance, Cui et al. (2022) evaluated the algal community in Luxun Park in Shanghai and concluded that the dominant Cyanobacteria accounted for over 60% in autumn [44]. Cyanobacteria generally have a broad temperature tolerance range and flourish during warm seasons [45]. The more abundant cyanobacteria in the aquatic habitats prompted the airborne cyanobacteria and further increased its concentration in HVAC, explaining the high concentration during autumn. Apart from that, some factors shape the cyanobacteria community. For instance, Wiśniewska et al. (2022) found that the number of microalgae and cyanobacteria cells decreased by up to 87% after a rainfall event relative to before the rainfall event [13]. Anthropogenic activities such as regularly cleaning the HVAC filters may reduce the microbiome in the filters. Those can explain the variation of cyanobacteria relative abundance in specific samples.

The cyanobacterial community structure varied significantly. The dust from HVAC filters was dominated by norank_chloroplast. This phenomenon remains a puzzle. We speculated that it lacks enough information and needs further identification, although the cyanobacteria are closely linked to the chloroplast from the evolutionary perspective. Several studies reported that norank_chloroplast could be suspended in the air or adhere to airborne fine particles. Similarly, the proportion of genera named after norank or unclassified cyanobacteriales also needs further identification.

We also detected many specific taxa, such as Chroococcidiopsaceae, Nostocaceae, Microcystaceae, and others in HVAC, which have also been reported in the atmosphere. Chroococcidiopsaceae can survive on stone surfaces [45] and usually were detected in biocrust [46,47]. Some biocrust-associated genera, such as Nostocaceae [46], were also detected. One view pointed out that airborne cyanobacteria originated from the soil [21]. We hold that not only the soil but also the aquatic sources produced the proportion of airborne cyanobacteria. Subsequently, the airborne cyanobacteria further deposited in the HVAC filters, in that the variation of cyanobacteria concentration followed a seasonal algal bloom pattern. The phytoplankton community responses have shown significant differences over seasonal and inter-annual time scales [48], and cyanobacteria tend to be dominant in autumn [41,48].

In addition, we detected some cyanobacteria that are usually found in the aquatic environment, indirectly indicating the contribution from the aquatic source. For instance, *Nodularia* sp., one of the principal genera causing the cyanobacterial blooms, were most commonly observed in the marine ecosystem, e.g., along the Andaman and Nicobar Islands in India [49] and the Baltic Sea [50]. As mentioned above, there are many inland ponds, rivers, and lakes, which are usually influenced by cyanobacteria growth and even algal bloom, located in Shanghai. Shanghai is also close to the sea, suggesting aquatic cyanobacteria escape from the water to the atmosphere.

From the perspective of potential toxins, the aerosolized cyanobacteria-associated health risk aroused public attention. For example, Facciponte et al. (2018) surveyed the cyanobacteria distribution in the human respiratory tract and concluded that the aerosol was an essential route for cyanobacteria exposure and a likely cause of cyanobacteria-associated human disease [15]. We detected some genera with a low relative abundance, and the obtained genera may be the harmful taxa, such as *Nodularia* sp., *Pseudanabaena* sp. and *Leptolyngbya* sp. [13,51]. The species, e.g., spumigena belonging to Nodularia, is a nuisance [52], as they can produce nodularin, a potent protein phosphatase inhibitor [50]. The Pseudanabaena can exist in terrestrial habitats and often occurs in planktonic and benthic communities of marine and freshwater ecosystems [53]. Some genera of Pseudanabaena can produce microcystins, anatoxins, and cylindrospermopsins [53]—the species, such as *Leptolyngbya* sp. RBD05, were toxic and showed the presence of algal microcystin [54]. The results illustrated that parts of species were potentially toxic, which should arouse attention regarding how to reduce the potential risk in the household from the HVAC system.

The present work compared the cyanobacterial community from HVAC following a season change. By reviewing the contemporary references, we found similar or different results in which the location could influence the profile of cyanobacteria. Sibanda et al. (2021) found cyanobacteria, accounting for the second major bacterial taxa in South Africa [55]. Noris et al. (2011) stated that the bacterial rank was Proteobacteria, Actinobacteria, and Firmicutes, according to their relative abundances in the southern U.S. [24]. The bias of geographic sites led to microbiological community changes and made us further explore the effects of cyanobacteria distribution in the dust of HVAC by the geographic variation in the future.

## 5. Conclusions

In conclusion, the contemporary works on cyanobacteria in habitats apart from the aquatic environment were limited. The present work studied the cyanobacteria concentration and community structure by the culture-independent approach of qPCR and high throughput sequencing technologies, providing much information on cyanobacterial taxa, which contributed to understanding the cyanobacteria distribution on the pre-filter of heating, ventilation, and air-conditioning (HVAC) systems. The findings demonstrated that the highest cyanobacterial DNA concentration occurred in autumn and occurred recurrently in Shanghai, with median values of 1,164.17 and 884.62 copies/cm^2^ in 2021 and 2022, respectively. Low median values of 394.21 for winter 2021 and 96.93 for summer 2022 copies/cm^2^ were found. The outdoor aquatic algal bloom by wave breaks, bubble bursts, and soil ascending by the wind could be the cyanobacteria source of the indoor HVAC. Chroococcidiopsaceae, norank_cyanobacteriales, Nostocaceae, and Paraspirulinaceae dominated the Cyanobacteria community in the dust from HVAC. Some detected genera, including *Nodularia* sp., *Pseudanabaena* sp., and *Leptolyngbya* sp., potentially produced cyanobacterial toxins. Thus, further assessment of potential threats to human health is necessary.

## Data Availability

The data presented in this study are available upon request from the corresponding author.
